# Prevalence and trends of cigarette smoking among adults with HIV infection compared with the general population in Korea

**DOI:** 10.4178/epih.e2024097

**Published:** 2024-12-16

**Authors:** Boyoung Park, Yoonyoung Jang, Taehwa Kim, Yunsu Choi, Kyoung Hwan Ahn, Jung Ho Kim, Hye Seong, Jun Yong Choi, Hyo Youl Kim, Joon Young Song, Shin-Woo Kim, Hee Jung Choi, Dae Won Park, Young Kyung Yoon, Sang Il Kim

**Affiliations:** 1 Department of Preventive Medicine, Hanyang University College of Medicine, Seoul, Korea; 2 Institute of Bioscience and Biotechnology, Hanyang University, Seoul, Korea; 3 Department of Agricultural Economics and Rural Development, Seoul National University, Seoul, Korea; 4 Department of Psychology, Sungkyunkwan University, Seoul, Korea; 5 Division of Infectious Diseases, Department of Internal Medicine, Yonsei University College of Medicine, Seoul, Korea; 6 AIDS Research Institute, Yonsei University College of Medicine, Seoul, Korea; 7 Division of Infectious Diseases, Department of Internal Medicine, Korea University College of Medicine, Seoul, Korea; 8 Division of Infectious Diseases, Department of Internal Medicine, Yonsei University Wonju College of Medicine, Wonju, Korea; 9 Department of Internal Medicine, Kyungpook National University School of Medicine, Deagu, Korea; 10 Department of Internal Medicine, Ewha Womans University College of Medicine, Ewha Womans University Mokdong Hospital, Seoul, Korea; 11 Division of Infectious Disease, Department of Internal Medicine, Seoul St. Mary’s Hospital, College of Medicine, The Catholic University of Korea, Seoul, Korea

**Keywords:** Human immunodeficiency virus, Tobacco smoking, Smoking cessation

## Abstract

**OBJECTIVES:**

This study compared the current smoking prevalence among adults with human immunodeficiency virus (HIV) infection to that of the general Korean population and analyzed changes in smoking prevalence and cessation rates from 2009 to 2020.

**METHODS:**

The study included a total of 10,980 adults with HIV infection who underwent a health screening examination (National Health Insurance Service-National Health Information Database; NHIS-NHID), 1,230 individuals with HIV infection who participated in the Korea HIV/AIDS Cohort (KoCosHIV), and 76,783 participants from the Korea National Health and Nutrition Examination Survey (KNHANES). We estimated the current smoking prevalence and the quit ratio, defined as the ratio of former smokers to ever-smokers.

**RESULTS:**

In the NHIS-NHID and KoCosHIV studies, the prevalence of current and former smoking among adults with HIV was 44.2% (95% confidence interval [CI], 43.2 to 45.1) and 15.6% (95% CI, 14.9 to 16.3), and 47.7% (95% CI, 43.7 to 51.8) and 16.9% (95% CI, 11.8 to 22.0), respectively. In the KNHANES, these rates were 22.5% and 18.1%, respectively. The standardized prevalence ratio of current smoking among adults with HIV was 1.76 in the NHIS-NHID and 1.97 in the KoCosHIV. Furthermore, the likelihood of quitting smoking was lower among adults with HIV than in the general population (NHIS-NHID: 26.1%; 95% CI, 25.0 to 27.1; KoCosHIV: 26.2%; 95% CI, 20.2 to 32.1; KNHANES: 44.6%; 95% CI, 44.5 to 44.6). Among HIV-positive adults, there was a 1.53% decline in the current smoking rate and a 2.86% increase in the quit ratio.

**CONCLUSIONS:**

Adults with HIV were more likely to smoke and less likely to quit smoking than the general adult population. Tobacco screening and cessation strategies should specifically target this population.

## INTRODUCTION

Despite a significant global decline in cigarette smoking since 1990, particularly in countries with high socio-demographic indexes, the global adult smoking prevalence was estimated at 32.6% for male and 6.5% for female in 2020, contributing to a substantial health burden [1]. Cigarette smoking remains the leading cause of death worldwide, responsible for 7 million deaths globally in 2020 [1]. In Korea, the prevalence of adult cigarette smoking decreased from 71.7% for male and 6.5% for female in 1992, to 39.7% for male and 3.3% for female in 2016, still indicating a high prevalence [2]. Cigarette smoking is linked to increased mortality due to non-human immunodeficiency virus (HIV)-related diseases such as cardiovascular diseases and non-acquired immunodeficiency syndrome (AIDS)-defining malignancies, as well as HIV-related mortality among individuals undergoing antiretroviral therapy [3–5]. Although more lives have been lost to cigarette smoking than to HIV even among adults with HIV infection [3], research indicates that the prevalence of current smoking is 2–3 times higher and the likelihood of quitting lower among adults with HIV infection than in the general population [6–8]. Thus, assessments of smoking status and intervention programs to support smoking cessation must be integrated into standard HIV care [9,10]. Various approaches to smoking cessation programs specifically targeting adults with HIV infection have been explored in clinical settings [11,12].

Despite the well-documented prevalence of smoking, its health effects, and trials for smoking cessation among Western populations with HIV, there has been limited research on smoking among adults with HIV in Asian countries. Korea, in particular, exhibits unique characteristics of HIV infection. It has seen an increase in newly diagnosed cases, predominantly among younger individuals (over 60% of these cases are among those aged 20 to 39), and a significantly higher proportion of these cases are in male (over 90% of new infections) [3,13]. Considering the distinct social contexts surrounding smoking in Asia, it is essential to investigate the smoking status of individuals with HIV in these regions.

Therefore, this study examined the prevalence of cigarette smoking and the quit ratio among adults with HIV infection compared to the general population. It utilized data from a nationwide population-based claims database and a multi-center HIV/AIDS cohort study in Korea. The research aimed to track changes in smoking prevalence and quit ratios among adults living with HIV and the general Korean population. This information will help inform prevention and care programs, including smoking cessation initiatives, for HIV-positive individuals.

## MATERIALS AND METHODS

### Data source

We utilized data from the National Health Insurance Service-National Health Information Database (NHIS-NHID), Korea HIV/AIDS Cohort Study (KoCosHIV), and Korea National Health and Nutrition Examination Survey (KNHANES) to estimate the prevalence of cigarette smoking among adults with HIV infection and the general Korean population. The NHIS, as the sole mandatory medical insurer in Korea, covers all residents and provides data on medical service utilization and health examination outcomes to researchers and policymakers via the NHIS-NHID. This database includes information on socio-demographic factors, healthcare service usage based on fee-for-service, prescribed medication, results of national health and cancer screenings, and vital status [14]. In the NHIS-NHID for the years 2004 to 2020, newly diagnosed adults with HIV infection aged ≥20 years were identified by a combination of the diagnosis codes for HIV infection (International Classification of Diseases 10th revision [ICD-10] codes of B20–B24) and the cost-sharing system code. This system supports treatment costs for conditions requiring significant medical expenditures, such as HIV treatment. For the years 2002–2003, individuals with healthcare service records using ICD-10 codes for HIV were excluded to ensure the identification of newly diagnosed cases, aligning with a study [15] that reported 16,671 new HIV infections from 2004 to 2020. National Health Screenings are available to all employees who pay insurance premiums, regardless of age, and to dependents aged 40 years or older who do not pay an annual or biennial premium. During these health examinations, health behaviors, including smoking status, are collected through a self-administered questionnaire. Consequently, of the 16,671 adults with HIV infection aged 20 years and older, 11,079 (66.4%) underwent national health screening between 2004 and 2020. After excluding 99 individuals due to missing smoking information, we included 10,980 adults with HIV infection in our analysis. We received authorization to analyze the pseudonymized data from NHIS-NHID, and therefore, the requirement for informed consent was waived.

The KoCosHIV was established in 2006 to support evidence-based prevention, treatment, and effective management of patients infected with HIV. Twenty-one tertiary hospitals across Korea participated in this initiative. The cohort’s inclusion criteria were adults aged 18 years or older who had a confirmed HIV infection by Western blot, were registered at the Korea Disease Control and Prevention Agency, and had voluntarily provided informed consent to participate in the study after receiving a comprehensive explanation from physicians. Participants underwent face-to-face interviews and had their blood samples collected by trained nurses. Follow-up examinations were conducted every 6 months. The KoCosHIV has been previously described in detail [16]. Of the 1,595 participants in the KoCosHIV, 1,230 adults with HIV infection (77.1%) who had available information on their smoking status at the time of recruitment were included in the study.

The KNHANES is a nationwide cross-sectional survey that annually includes approximately 10,000 individuals using a multi-stage clustered probability design. It targets non-institutionalized individuals aged 1 year and older. The survey consists of a health interview, which includes questions about smoking status, as well as a physical and biochemical examination, and a nutrition survey, all conducted by trained staff. The methodology of the KNHANES has been extensively described elsewhere [17]. From 2009 to 2020, the KNHANES included 98,753 participants, of whom 76,783 aged 20 years and older had available information on their smoking status. The data from the 2009–2020 KNHANES were weighted to adjust for the sample design, differential probability of selection, and nonresponse, ensuring they represent the Korean population accurately.

### Measurement of smoking status

The primary outcome was the current smoking status. The following questions were asked:

- “Have you smoked at least 5 packs of cigarettes (100 cigarettes) during your lifetime?” (NHIS-NHID, KoCosHIV);
- “How many packs (or number) of cigarettes have you smoked throughout your life: none, <5 packs (100 cigarettes), and ≥5 packs (100 cigarettes)?” (KNHANES); and
- “Do you currently smoke?” (NHIS-NHID, KoCosHIV, KNHANES).

Current smokers were defined as individuals who had smoked at least 5 packs of cigarettes in their lifetime and continued to smoke at the time of the study. Former smokers were those who had smoked at least 5 packs of cigarettes in their lifetime but had quit by the time of the study. The ratio of former smokers to ever-smokers (both current and former smokers) was defined as the “quit ratio,” in line with previous studies [6,7]. The smoking status of adults with HIV infection from the NHIS-NHID was determined based on the most recent smoking status recorded at the time of their health screening since the date of HIV diagnosis. In the KoCosHIV, smoking status was assessed at baseline. Smoking status for each year was estimated through information gathered from repeated health screenings, conducted annually or biennially as part of the NHIS-NHID, or from follow-up questionnaires as part of the KoCosHIV. In the NHIS-NHID, the questionnaire regarding the amount and duration of smoking was revised in 2009. Although this study did not consider the duration or extent of smoking, trends in smoking status and the quit ratio were observed from 2009 to 2020.

### Demographic variables

The demographic variables available in the NHIS-NHID included sex, age, and health insurance type (National Health Insurance and Medical Aid), all of which were obtained through administrative data. In the KoCosHIV, demographic information such as sex, age, marital status, occupation, educational level, income, and medical insurance type was collected via a questionnaire. Variables common to both NHIS-NHID and KoCosHIV, including sex, age, and medical insurance type, were analyzed. Additionally, current alcohol drinking status and average drinking frequency of at least once a month over the previous year were assessed. These data were collected in the KNHANES using a questionnaire.

### Statistical analysis

In the KNHANES data, the weighted proportion and 95% confidence interval (CI) of current and former smokers were calculated to represent the smoking status of the Korean adult population. Estimates were made for both the pooled weighted proportion of smoking status and the annual prevalence of current smoking from 2009 to 2020. Additionally, based on the numbers of former and current smokers, estimates were made for the overall and annual quit ratios during the same period. In the NHIS-NHID and KoCosHIV datasets, the proportion and 95% CI of current smokers and the quit ratio were calculated using the most recent data from either the HIV diagnosis or the baseline cohort survey. Using repeated measures of smoking status, the prevalence of current smoking and the quit ratio for each year from 2009 to 2020 were estimated. The study also presented differences in the prevalence of current smoking and quit ratios between adults with HIV infection and the general Korean adult population, stratified by demographic variables. The standardized prevalence ratio (SPR) for current smoking was calculated using age groups in 5-year increments and by sex. Additionally, the SPR was stratified by sex using the same age grouping. To quantify the percentage changes in the prevalence of current smoking and quit ratios from 2009 to 2020, the annual percent change (APC), defined as a summary measure of the trend over a pre-specified fixed interval, of the prevalence of current smoking and quit ratio was estimated based on the linear regression model as {exp(β)−1}×100 [18]. Statistical analyses were performed using the SAS, version 9.4 (SAS Institute Inc., Cary, NC, USA). The p-values were reported as 2-tailed, and statistical significance was set at p<0.05.

### Ethics statement

This study received approval from the Institutional Review Board of Hanyang University, Korea (approval No. HYUIRB-202111-005).

## RESULTS

The final analysis included 10,980 NHIS-NHID participants, 1,230 KoCosHIV participants, and 76,783 KNHANES participants, as shown in Table 1. In the NHIS-NHID and KoCosHIV cohorts, adults with HIV infection were predominantly male (≥90%) and most were over 50 years of age (approximately 70% or more). The KNHANES sample, representing 40,122,739 non-institutionalized adults living in Korea, was evenly distributed between male and female, with 57.7% of the population under the age of 50.

Table 2 displays the prevalence of current smoking among adults with HIV infection compared to Korean adults in the general population. In the NHIS-NHID and KoCosHIV datasets, the prevalence of current smoking among adults with HIV infection was 44.2% (95% CI, 43.2 to 45.1) and 47.7% (95% CI, 43.7 to 51.8), respectively. The prevalence of former smoking was 15.6% (95% CI, 14.9 to 16.3) and 16.9% (95% CI, 11.8 to 22.0) in the NHIS-NHID and KoCosHIV, respectively (data not shown). In contrast, in the general Korean adult population, 22.5% (95% CI, 22.5 to 22.5) were current smokers and 18.1% (95% CI, 18.1 to 18.1) were former smokers. The prevalence of current smoking was notably higher among adults with HIV infection than in the general population. Additionally, the prevalence of current smoking was higher among and current alcohol users in both the HIV-infected and general populations. After the age of 50, the prevalence of current smoking decreased with increasing age in both groups.

After adjusting for age and sex distributions between adults with HIV infection and the general population using indirect standardization, we found that current smoking was more prevalent among adults with HIV infection than in the general population, particularly among female in both datasets. The prevalence ratio (SPR) of current smoking was 1.76 (95% CI, 1.71 to 1.81) in the NHIS-NHID and 1.97 (95% CI, 1.81 to 2.13) in the KoCosHIV, compared to the general population (Table 3). In the NHIS-NHID and KoCosHIV, adult male with HIV infection had SPRs of 1.10 (95% CI, 1.07 to 1.14) and 1.20 (95% CI, 1.10 to 1.30), respectively. For female with HIV infection, the SPRs were significantly higher, at 1.85 (95% CI, 1.50 to 2.20) in the NHIS-NHID and 2.92 (95% CI, 1.44 to 4.40) in the KoCosHIV. From 2009 to 2020, despite yearly variations in the SPR, current smoking remained consistently more prevalent among adults with HIV infection across nearly all the years observed.

Table 4 presents the quit ratio among adults with HIV infection compared to Korean adults in the general population. Adults with HIV infection were less likely to quit smoking than their counterparts in the general population. The quit ratio for ever-smokers with HIV infection was 26.1% (95% CI, 25.0 to 27.1) in the NHIS-NHID and 26.2% (95% CI, 20.2 to 32.1) in the KoCosHIV. In contrast, the quit ratio among Korean adults in the general population was 44.6% (95% CI, 44.5 to 44.6). The quit ratios among adults with HIV infection were lowest in the 20–29 age group, approximately 16.0% in both the NHIS-NHID and KoCosHIV, and increased with age, reaching 62.3% and 75.0% among those aged ≥70 years in the NHIS-NHID and KoCosHIV, respectively. Across all considered characteristics, adults with HIV infections exhibited lower quit ratios than the general Korean adult population.

**Figure 1:**
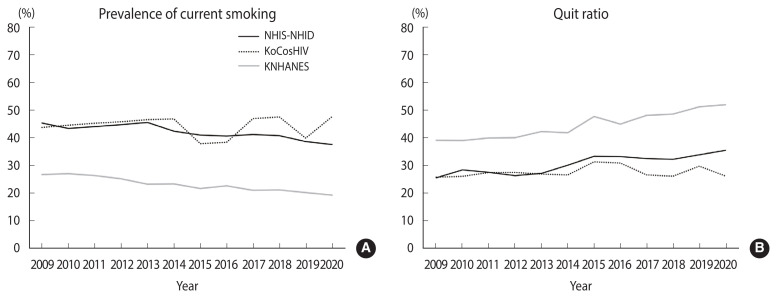
Annual prevalence of smoking and quit ratio among adults with HIV infection and among the general population in Korea from 2009 to 2020. (A) Prevalence of current smoking. The APC for yearly changes was −1.53% (95% CI, −2.04 to −1.02; p<0.001) in the NHIS-NHID, −0.13% (95% CI, −1.74 to 1.51; p=0.862) in the KoCosHIV, and −3.03% (95% CI, −3.50 to −2.56; p<0.001) in the KNHANES. (B) Quit ratio. The APC for yearly changes was 2.86% (95% CI, 1.92 to 3.80; p<0.001) in the NHIS-NHID, 0.50% (95% CI, −0.80 to 1.80; p=0.415) in the KoCosHIV, and 2.89% (95% CI, 2.33 to 3.46; p<0.001) in the KNHANES. HIV, human immunodeficiency virus; NHIS-NHID, adults with HIV infection identified from the National Health Insurance Service-National Health Information Database; KoCosHIV, Korea HIV/AIDS Cohort; KNHANES, Korea National Health and Nutrition Examination Survey; APC, annual percent change.

Figure 1 illustrates the trends in the prevalence of current smoking and the quit ratio from 2009 to 2020. The prevalence of current smoking in the NHIS-NHID decreased from 45.3% (95% CI, 43.4 to 47.3) in 2009 to 37.6% (95% CI, 36.0 to 39.2) in 2020, with an APC of −1.53% (p<0.001). In contrast, the KoCosHIV data showed that the prevalence of current smoking was 43.8% (95% CI, 39.6 to 47.8) in 2009 and 47.7% (95% CI, 43.6 to 51.7) in 2020, indicating no significant changes (p=0.862). Among the general Korean adult population, the prevalence of current smoking declined from 26.8% (95% CI, 26.8 to 26.8) in 2009 to 19.3% (95% CI, 19.3 to 19.4%) in 2020, with an APC of −3.03% (p<0.001). The quit ratios showed an increase each year. In the NHIS-NHID, the quit ratio rose from 25.6% (95% CI, 21.2 to 30.1) in 2009 to 35.6% (95% CI, 32.2 to 39.0) in 2020, with an APC of 2.86% (p<0.001). In the KoCosHIV, the quit ratio slightly increased from 25.9% (95% CI, 19.9 to 31.8) in 2009 to 26.3% (95% CI, 20.3 to 32.2) in 2020, with an APC of 0.50% (p=0.415). In the KNAHNES, the quit ratio improved from 39.2% (95% CI, 39.2 to 39.3) in 2009 to 52.1% (95% CI, 52.0 to 52.1) in 2020, with an APC of 2.89% (p<0.001).

## DISCUSSION

The current smoking prevalence among adults with HIV infection, measured during the health screening closest to their HIV diagnosis date, ranged from 44.2% to 47.7%. This rate is 1.7 to 2.0 times higher than the pooled current smoking prevalence of 21.7% observed in the general Korean adult population between 2009 and 2020, even after adjusting for age and sex. Additionally, adults with HIV were less likely to quit smoking compared to their counterparts in the general Korean population, with quit rates of approximately 26.0% versus 44.6%. Studies from Western countries have also reported a higher smoking prevalence among adults with HIV compared to the general population, findings that align with those observed in Korea [6–8]. The current smoking prevalence among adults with HIV in Korea is comparable to that in the United States [6,8] and Denmark [19], but lower than that of their counterparts in Italy [20].

Although adults with HIV infection demonstrated a higher current smoking prevalence, their demographic patterns were comparable to those of the general population. A higher prevalence of smoking was observed among male aged 30–39 years and among current alcohol users. However, when considering all demographic factors, adults with HIV infection exhibited a higher prevalence of current smoking than the general population. This finding is consistent with the results of a previous study [6]. When stratified by sex, the current smoking prevalence among male with HIV infection in Korea was approximately 50%, similar to previous studies, while it ranged from 10–18% among female with HIV infection, which is lower than reported in other studies [6,21]. A meta-analysis found the current smoking prevalence to be 50.3% among male and 36.3% among female with HIV infection. However, the studies included in the meta-analysis showed that smoking prevalence varied widely, ranging from 6.2% to 91.2% among male and from 0.0% to 90.0% among female [21]. In this meta-analysis, the current smoking prevalence among male was 1.78 times higher than among female. Studies focusing solely on the United States have indicated a similar smoking prevalence between male and female, at ≥50%. Given the lower smoking prevalence among female compared to male in the general population, a similar prevalence level among both sexes for Americans with HIV infection suggests that HIV infection is associated with a larger discrepancy in the smoking rate for female than for male [21]. In Korea, among adults with HIV infection, the prevalence of current smoking was 3–4 times higher among male than among female, reflecting a larger sex gap than that observed in other countries.

The prevalence of current smoking decreased and the quit ratio increased among both adults with HIV infection and the general population, according to data from the NHIS-NHID and KNHANES for the years 2009 and 2020. Previous studies have documented this trend [7,8]. In the KoCosHIV, however, both smoking prevalence and quit ratio showed no significant annual variation. The small sample size of the KoCosHIV may have contributed to the statistically non-significant APC in yearly data. The KoCosHIV is an open cohort, and the number of participants receiving follow-up care varied each year. Despite the statistically significant decline in current smoking prevalence, the APC’s slope was steeper among the general population than among adults with HIV infection in Korea. Adults with HIV were less likely to quit smoking compared to the general population (approximately 26.0 vs. 44.6%), despite the increased quit ratio observed in both groups from the NHIS-NHID and KNHANES data. These findings suggest that national smoking cessation programs and various other quitting initiatives over recent decades in Korea [22,23] may have been less effective for adults with HIV infection. Studies have linked the higher prevalence of smoking and lower quit ratio among adults with HIV infection to factors such as low socioeconomic status, ethnic disparities, disparities in mental health diagnoses, and other risk factors associated with HIV infection [24]. Although we could not compare the socioeconomic status of adults with HIV infection directly with that of the general population in Korea, the proportion of individuals supported by medical aid programs was similar between both groups, suggesting a potential independent association between HIV infection and smoking habits. Biological mechanisms, including the rapid metabolism of nicotine among smokers with HIV infection [25], which is associated with severe nicotine dependence and subsequent difficulty in quitting smoking [26], could be contributing to the higher smoking prevalence and lower quitting ratios. The antiretroviral treatment efavirenz, used in adults with HIV infection, has been associated with accelerated nicotine metabolism [27,28], further complicating smoking cessation efforts even among those receiving proper treatment. Additionally, studies have shown reduced effectiveness of pharmacotherapies for smoking cessation among adults with HIV infection [29,30]. Although we could not directly assess the causes of the high current smoking prevalence and low quit ratio among adults with HIV infection in Korea, these mechanisms likely explain the findings of this study.

This study has several limitations. First, smoking status was assessed through self-reported answers, which may not accurately reflect the actual smoking habits of participants due to the stigma associated with smoking, particularly among female in Asian countries. A study comparing self-reported smoking status with urine cotinine levels in the Korean population found that the low smoking rates reported were similar to those estimated from urinary cotinine levels, with even lower rates observed among female [31]. Given the small proportion of female among adults with HIV infection (<10%), the overall smoking prevalence for both male and female in the general population is likely more influenced by the underreporting of smoking rates than by HIV status. However, when analyzed by sex, the smoking prevalence was higher among adults with HIV infection for both sexes. Within the same sex, the underestimation of smoking status based on HIV infection status could lead to a non-differential bias, making the observed association appear weaker [32]. Second, the adults with HIV infection identified from the NHIS-NHID or the KoCosHIV were participants in a health screening program or a multi-center cohort in Korea. Therefore, the estimates may not reflect the smoking prevalence and quit ratios among those who did not participate in health screenings or the cohort, or among those with undiagnosed HIV infections. Third, the KNHANES population might have included individuals with diagnosed or undiagnosed HIV infections. However, the prevalence of HIV among the NHIS population is likely negligible, given the overall HIV prevalence in the Korean population (0.03%). Fourth, there may be an overlap between the population with HIV infection from the NHIS-NHID and that from the KoCosHIV. However, since we used pseudonymized data from both the NHIS-NHID and KoCosHIV, we could not determine the extent of this overlap. Nevertheless, the similarity in results from both datasets lends greater reliability to our findings. Fifth, in comparing the smoking prevalence among adults with HIV infection to that of the general population, we only adjusted for age and sex distributions and did not account for other characteristics that might influence smoking status. Future analytical epidemiological studies with larger sample sizes should be conducted to determine whether smoking prevalence is higher or lower among adults with HIV infection compared to the general population or to individuals without HIV infection, after controlling for other confounding variables associated with both smoking status and HIV infection.

In conclusion, the current smoking prevalence among adults with HIV infection was higher than that of the general Korean adult population, even after adjusting for sex and age. Additionally, the quit ratio was lower among adults with HIV infection compared to Korea’s general population. Despite a decrease in current smoking prevalence, the rate of decline was slower among adults with HIV infection than among Korean adults in the general population, indicating growing disparities in smoking rates between these groups. Although quit ratios improved between 2009 and 2020, adults with HIV were still less likely to quit smoking than their counterparts in the general Korean population. This highlights the urgent need for smoking cessation programs specifically designed for individuals with HIV. While several trials have aimed to develop targeted smoking cessation programs for adults with HIV infection, no such trials have yet been conducted in Korea. Developing tailored smoking cessation interventions that consider the Korean context and provide expanded access to preventive medical services, including smoking cessation for adults with HIV infection, could help reduce smoking rates and improve quit ratios, ultimately decreasing morbidity and mortality associated with smoking in these vulnerable populations.

## Figures and Tables

**Table 1:** Characteristics of adults with HIV infection in Korea identified by the NHIS-NHID, participants in the KoCosHIV, and general population from the KNHANES

Characteristics	NHIS-NHID		KoCosHIV		KNHANES			
	n	% (95% CI)	n	% (95% CI)	n	% (95% CI)	Weighted n	Weighted % (95% CI)
Total	10,980	100	1,230	100	76,783	100	40,122,739	100
Sex								
Male	9,915	90.3 (89.8, 90.8)	1,146	93.2 (91.8, 94.6)	33,630	43.8 (43.5, 44.2)	19,906,741	49.6 (49.6, 49.6)
Female	1,065	9.7 (9.2, 10.3)	84	6.8 (5.4, 8.2)	43,153	56.2 (55.8, 56.5)	20,215,998	50.4 (50.4, 50.4)
Age (yr)								
Mean±SD		42.4±13.4		40.7±012.1		51.2±16.6	-	-
20–29	2,214	20.2 (19.4, 20.9)	254	20.6 (18.4, 22.9)	8,822	11.5 (11.3, 11.7)	6,890,092	17.2 (17.2, 17.2)
30–39	2,631	24.0 (23.2, 24.8)	336	27.3 (24.8, 29.8)	12,959	16.9 (16.6, 17.1)	7,767,767	19.4 (19.4, 19.4)
40–49	2,807	25.6 (24.7, 26.4)	342	27.8 (25.3, 30.3)	14,161	18.4 (18.2, 18.7)	8,477,697	21.1 (21.1, 21.1)
50–59	1,983	18.1 (17.3, 18.8)	207	16.8 (14.7, 18.9)	14,516	18.9 (18.6, 19.2)	7,750,003	19.3 (19.3, 19.3)
60–69	955	8.7 (8.2, 9.2)	75	6.1 (4.8, 7.4)	13,196	17.2 (16.9, 17.5)	4,876,043	12.2 (12.1, 12.2)
≥70	390	3.6 (3.2, 3.9)	16	1.3 (0.7, 1.9)	13,129	17.1 (16.8, 17.4)	4,361,137	10.9 (10.9, 10.9)
Income								
Medical Aid	366	3.3 (3.0, 3.7)	128	10.4 (8.7, 12.1)	2,738	3.6 (3.4, 3.7)	38,517,406	96.0 (96.0, 96.0)
NHI	10,610	96.6 (96.3, 97.0)	716	58.2 (55.5, 61.0)	74,000	96.4 (96.2, 96.5)	1,244,731	3.1 (3.1, 3.1)
None	4	0.0 (0.0, 0.1)	386	31.4 (28.8, 34.0)	45	0.1 (0.0, 0.1)	360,602	0.9 (0.9, 0.9)
Alcohol use								
Never/past	5,027	45.8 (44.8, 46.7)	514	41.8 (39.0, 44.5)	33,501	43.6 (43.3, 44.0)	20,385,583	50.8 (50.8, 50.8)
Current	5,858	53.4 (52.4, 54.3)	622	50.6 (47.8, 53.4)	37,323	48.6 (48.3, 49.0)	18,429,730	45.9 (45.9, 46.0)
Unknown	95	0.9 (0.7, 1.0)	94	7.6 (6.2, 9.1)	5,959	7.8 (7.6, 7.9)	1,307,426	3.3 (3.2, 3.3)

HIV, human immunodeficiency virus; NHIS-NHID, National Health Insurance Service-National Health Information Database; KoCosHIV, Korea HIV/AIDS Cohort; KNHANES, Korea National Health and Nutrition Examination Survey; CI, confidence interval; SD, standard deviation; NHI, National Health Insurance.

**Table 2:** Prevalence of current smoking among adults with HIV infection in Korea identified by the NHIS-NHID, participants in the KoCosHIV, and general population from the KNHANES

Characteristics	NHIS-NHID	KoCosHIV	KNHANES	Prevalence difference	
	% (95% CI)	% (95% CI)	Weighted % (95% CI)	NHIS-NHID–KNAHNES %p (95% CI)	KoCosHIV-KNHANES %p (95% CI)
Total	44.2 (43.2, 45.1)	47.7 (43.7, 51.8)	22.5 (22.5, 22.5)	21.7 (20.8, 22.6)	25.2 (21.2, 29.3)
Sex					
Male	47.8 (46.2, 48.8)	49.9 (45.9, 54.0)	39.4 (39.4, 39.4)	8.4 (7.4, 9.4)	10.5 (6.5, 14.5)
Female	10.2 (8.4, 12.1)	17.9 (14.8, 21.0)	5.8 (5.8, 5.8)	4.4 (2.6, 6.2)	12.0 (9.0, 15.1)
Age (yr)					
20–29	48.2 (46.2, 50.3)	48.8 (44.8, 52.9)	26.1 (26.1, 26.1)	22.1 (20.1, 24.2)	22.7 (18.7, 26.7)
30–39	50.1 (48.2, 52.0)	52.1 (48.0, 56.1)	28.9 (28.8, 28.9)	21.3 (19.4, 23.1)	23.2 (19.2, 27.2)
40–49	49.7 (47.9, 51.6)	54.1 (50.1, 58.1)	25.6 (25.6, 25.6)	24.1 (22.3, 26.0)	28.5 (24.5, 32.5)
50–59	38.6 (36.5, 40.8)	39.1 (35.2, 43.1)	21.4 (21.4, 21.5)	17.2 (15.1, 19.3)	17.7 (13.8, 21.6)
60–69	26.3 (23.5, 29.1)	26.7 (23.1, 30.2)	15.5 (15.4, 15.5)	10.8 (8.1, 13.6)	11.2 (7.7, 14.8)
≥70	12.6 (9.3, 15.9)	12.5 (9.8, 15.2)	9.0 (9.0, 9.1)	3.5 (0.3, 6.8)	3.5 (0.8, 6.1)
Income					
Medical Aid	43.7 (38.6, 48.8)	48.4 (44.4, 52.5)	27.2 (27.1, 27.3)	16.5 (11.5, 21.5)	21.2 (17.3, 25.2)
NHI	44.2 (43.2, 45.1)	48.2 (44.1, 52.2)	22.3 (22.3, 22.3)	21.9 (20.9, 22.8)	25.9 (21.8, 29.9)
Alcohol use					
Never/past	32.3 (31.0, 33.6)	38.7 (34.8, 42.7)	12.3 (12.3, 12.3)	20.0 (18.7, 21.3)	26.4 (22.5, 30.4)
Current	55.0 (53.7, 56.3)	60.9 (57.0, 64.9)	35.3 (35.3, 35.3)	19.7 (18.5, 21.0)	25.6 (21.7, 29.6)

HIV, human immunodeficiency virus; NHIS-NHID, National Health Insurance Service-National Health Information Database; KoCosHIV, Korea HIV/AIDS Cohort; KNHANES, Korea National Health and Nutrition Examination Survey; CI, confidence interval; NHI, National Health Insurance.

**Table 3:** Standardized prevalence ratio of current smoking and its trend among adults with HIV infection in Korea identified by the NHIS-NHID and participants in the KoCosHIV, compared with the general population from the KNHANES

Year	Adults with HIV infection from the NHIS-NHID			Adults with HIV infection from the KoCosHIV		
	Total	Male	Female	Total	Male	Female
Total	1.76 (1.71, 1.81)	1.10 (1.07, 1.14)	1.85 (1.50, 2.20)	1.97 (1.81, 2.13)	1.20 (1.10, 1.30)	2.92 (1.44, 4.40)
2009	1.64 (1.54, 1.74)	1.02 (0.96, 1.09)	1.30 (0.73, 1.87)	1.78 (1.53, 2.04)	1.09 (0.93, 1.25)	2.20 (0.27, 4.13)
2010	1.51 (1.42, 1.60)	0.92 (0.87, 0.99)	1.29 (0.68, 1.90)	1.70 (1.50, 1.89)	1.02 (0.90, 1.14)	2.80 (0.86, 4.73)
2011	1.60 (1.51, 1.70)	0.99 (0.93, 1.05)	1.01 (0.50, 1.52)	1.78 (1.60, 1.97)	1.06 (0.95, 1.17)	3.09 (1.26, 4.92)
2012	1.69 (1.60, 1.79)	1.07 (1.01, 1.12)	1.02 (0.54, 1.51)	1.85 (1.67, 2.03)	1.12 (1.01, 1.23)	2.85 (1.36, 4.35)
2013	1.82 (1.72, 1.92)	1.12 (1.06, 1.18)	1.74 (1.08, 2.40)	2.01 (1.82, 2.20)	1.18 (1.07, 1.29)	3.73 (1.78, 5.68)
2014	1.70 (1.61, 1.79)	1.01 (0.96, 1.07)	1.42 (0.75, 2.10)	2.00 (1.82, 2.18)	1.14 (1.04, 1.25)	4.53 (2.16, 6.90)
2015	1.78 (1.68, 1.87)	1.08 (1.02, 1.14)	1.68 (1.02, 2.33)	1.94 (1.75, 2.13)	1.15 (1.04, 1.27)	4.29 (2.04, 6.53)
2016	1.69 (1.60, 1.78)	1.06 (1.00, 1.12)	0.94 (0.49, 1.38)	1.88 (1.71, 2.06)	1.15 (1.04, 1.26)	3.07 (1.46, 4.68)
2017	1.85 (1.76, 1.95)	1.15 (1.09, 1.21)	1.69 (1.00, 2.38)	2.26 (2.07, 2.45)	1.34 (1.23, 1.46)	4.48 (2.13, 6.83)
2018	1.76 (1.67, 1.84)	1.13 (1.07, 1.18)	1.29 (0.78, 1.81)	2.22 (2.04, 2.41)	1.38 (1.26, 1.49)	3.38 (1.61, 5.15)
2019	1.79 (1.70, 1.88)	1.13 (1.07, 1.19)	1.90 (1.27, 2.53)	2.25 (2.05, 2.45)	1.37 (1.24, 1.49)	3.59 (1.71, 5.47)
2020	1.81 (1.71, 1.91)	1.14 (1.08, 1.21)	1.24 (0.65, 1.83)	2.54 (2.33, 2.74)	1.53 (1.40, 1.65)	3.92 (1.94, 5.91)

Values are presented as standardized prevalence ratio (95% confidence interval).

HIV, human immunodeficiency virus; NHIS-NHID, National Health Insurance Service-National Health Information Database; KoCosHIV, Korea HIV/AIDS Cohort; KNHANES, Korea National Health and Nutrition Examination Survey.

**Table 4:** Prevalence of quit ratio^1^ among adults with HIV infection in Korea identified by the NHIS-NHID, participants in the KoCosHIV, and general population from the KNHANES

Characteristics	NHIS-NHID	KoCosHIV	KNHANES	Prevalence difference	
	% (95% CI)	% (95% CI)	Weighted % (95% CI)	NHIS-NHID–KNAHNES %p (95% CI)	KoCosHIV-KNHANES %p (95% CI)
Total	26.1 (25.0, 27.1)	26.2 (20.2, 32.1)	44.6 (44.5, 44.6)	−18.5 (−19.5, −17.5)	−18.4 (−24.4, −12.5)
Sex					
Male	26.0 (24.9, 27.1)	26.2 (20.2, 32.2)	44.7 (44.6, 44.7)	−18.7 (−19.7, −17.6)	−18.48 (−24.4, −12.5)
Female	28.8 (21.6, 35.9)	25.0 (19.1, 30.9)	43.9 (43.8, 44.0)	−15.1 (−22.2, −8.0)	−18.88 (−24.7, −13.1)
Age (yr)					
20–29	16.0 (14.0, 18.0)	16.8 (11.7, 21.9)	24.4 (24.4, 24.5)	−8.5 (−10.4, −6.5)	−7.66 (−12.7, −2.6)
30–39	19.8 (17.8, 21.7)	22.2 (16.6, 27.9)	33.5 (33.5, 33.6)	−13.7 (−15.6, −11.9)	−11.29 (−16.9, −5.7)
40–49	26.3 (24.3, 28.3)	26.9 (20.9, 32.9)	41.7 (41.7, 41.8)	−15.4 (−17.3, −13.5)	−14.83 (−20.8, −8.9)
50–59	34.7 (32.1, 37.4)	34.2 (27.7, 40.6)	49.9 (49.9, 50.0)	−15.2 (−17.9, −12.5)	−15.75 (−22.2, −9.4)
60–69	43.9 (39.3, 48.5)	46.0 (39.2, 52.7)	62.4 (62.3, 62.5)	−18.6 (−23.1, −14.0)	−16.44 (−23.2, −9.7)
≥70	62.3 (54.0, 70.6)	75.0 (69.1, 80.9)	73.3 (73.2, 73.4)	−11.0 (−19.2, −2.7)	1.71 (−4.1, 7.5)
Income					
Medical Aid	25.9 (20.1, 31.8)	28.7 (22.6, 34.9)	38.8 (38.7, 38.9)	−12.9 (−18.6, −7.1)	−10.05 (−16.1, −4.0)
NHI	26.1 (25.0, 27.2)	25.4 (19.5, 31.4)	44.9 (44.9, 44.9)	−18.8 (−19.9, −17.8)	−19.46 (−25.4, −13.6)
Alcohol use					
Never/past	32.6 (30.8, 34.5)	36.0 (29.5, 42.5)	52.5 (52.4, 52.5)	−19.8 (−21.6, −18.0)	−16.44 (−22.9, −10.0)
Current	22.2 (21.0, 23.5)	19.7 (14.3, 25.1)	40.8 (40.7, 40.8)	−18.5 (−19.8, −17.3)	−21.07 (−26.4, −15.7)

HIV, human immunodeficiency virus; NHIS-NHID, National Health Insurance Service-National Health Information Database; KoCosHIV, Korea HIV/AIDS Cohort; KNHANES, Korea National Health and Nutrition Examination Survey; CI, confidence interval; NHI, National Health Insurance.

1 Quit ratio=number of former smokers/number of ever-smokers, including both current and former smokers.
